# Diagnostic value of metagenomic next generation sequencing of bronchoalveolar lavage fluid in immunocompromised patients with pneumonia

**DOI:** 10.3389/fcimb.2025.1602636

**Published:** 2025-09-16

**Authors:** Liu Xin, Xiaodan Jiao, Xiaowei Gong, Jing Yu, Jing Zhao, Jing Lv, Qixuan Feng, YaDong Yuan, Wensen Pan

**Affiliations:** Department of Pulmonary and Critical Care Medicine, The Second Hospital of Hebei Medical University, Shijiazhuang, China

**Keywords:** immunocompromised pneumonia, metagenomic sequencing, conventional microbiological testing, pathogen spectrum, diagnostic accuracy

## Abstract

**Background:**

Metagenomic next-generation sequencing (mNGS) enables simultaneous sequencing of DNA fragments for comprehensive pathogen identification. Pneumonia in immunocompromised patients—characterized by atypical clinical manifestations and rapid progression—poses diagnostic challenges. Conventional microbiological testing (CMT), which relies on pathogen culture and serological assays, is limited by prolonged turnaround times and suboptimal detection rates. This study was performed to evaluate the clinical utility of mNGS through comparative analysis with CMT in detecting pathogens among immunocompromised patients with pneumonia.

**Methods:**

We conducted a retrospective cohort study of 146 immunocompromised patients with suspected pneumonia. The mNGS and CMT results were systematically analyzed. Pathogen detection rates and microbial spectrum concordance were visualized using pie and bar charts. Diagnostic performance was compared using McNemar’s test and Kappa (κ) statistics for inter-method agreement. The sensitivity, specificity, accuracy, and area under the curve were calculated for pathogen-specific evaluations.

**Results:**

mNGS demonstrated superior detection efficacy, identifying pathogens in 98 cases versus 50 by CMT, with 48 overlapping positives. The microbial spectrum showed substantial differences: mNGS detected 73 bacterial, 46 fungal, and 45 viral pathogens, whereas CMT identified 38 bacterial, 27 fungal, and 21 viral agents. mNGS outperformed CMT across all infection types, including single-pathogen infections (bacterial, fungal, or viral only) and mixed infections (bacterial + fungal, bacterial + viral, fungal + viral, or bacterial + fungal + viral). Bacterial and fungal detections showed low inter-method concordance, while viral detection exhibited moderate agreement (κ = 0.510, *p* < 0.001). Notably, mNGS achieved significantly higher detection rates for *Enterococcus faecalis* and *Pneumocystis jirovecii* in intensive care unit (ICU)-admitted patients with severe pneumonia (*p* < 0.05). Clinical outcomes improved in 45 patients following mNGS-guided therapeutic adjustments.

**Conclusions:**

mNGS and CMT demonstrate complementary strengths in bacterial and fungal detection in immunocompromised patients with pneumonia. mNGS provides enhanced diagnostic accuracy for key pathogens such as *E. faecalis* and *P. jirovecii*, particularly in severe and ICU-admitted cases. As a high-throughput diagnostic tool, mNGS may improve pathogen detection and clinical management in immunocompromised populations.

## Background

Metagenomic next-generation sequencing (mNGS) is a high-throughput technology capable of simultaneously sequencing millions to billions of DNA fragments for unbiased pathogen identification (Gu et al., 2019). By eliminating polymerase chain reaction (PCR) amplification bias, this method enables rapid detection of diverse pathogens (bacteria, fungi, viruses, and parasites) across clinical specimens. Notably, mNGS addresses critical limitations of conventional microbiological testing (CMT), including prolonged turnaround times and suboptimal sensitivity, thereby emerging as a transformative tool for infectious disease diagnostics (Tsang et al., 2022). However, despite established technical guidelines, challenges persist in standardizing its clinical implementation and interpreting complex microbial profiles (Qian et al., 2021).

Immunodeficiency, characterized by impaired host defense mechanisms, significantly increases susceptibility to opportunistic infections (Murali et al., 2022). In immunocompromised pneumonia—a life-threatening complication associated with glucocorticoid therapy, hematopoietic stem cell transplantation, and congenital immune disorders—atypical presentations and rapid progression to multiorgan failure necessitate precise pathogen identification (Ramirez et al., 2020). Conventional diagnostic approaches often fail to provide timely results, underscoring the urgent need for advanced methodologies to guide targeted antimicrobial therapy and improve clinical outcomes.

Although mNGS has demonstrated diagnostic superiority in community-acquired pneumonia (CAP) cohorts, existing evidence remains limited for immunocompromised populations. Qin et al (Qin et al., 2024). reported that mNGS achieved 92.3% sensitivity for bacterial pathogen detection in 207 patients with CAP, outperforming culture-based methods. Similarly, Hu et al (Hu et al., 2024). documented the enhanced capacity of mNGS to identify atypical and fungal pathogens in 145 patients with severe pneumonia (area under the curve: 0.86 vs. 0.71 for CMT). However, no large-scale studies have systematically evaluated mNGS performance using bronchoalveolar lavage fluid (BALF) samples from immunocompromised patients with pneumonia. This study comprehensively compared mNGS and CMT in detecting bacterial, fungal, and viral pathogens, aiming to establish evidence-based protocols for optimizing diagnostic workflows in this high-risk population.

## Methods

### Study design and patient selection

From January 2020 to March 2023, 146 immunocompromised patients with suspected pneumonia from the Second Hospital of Hebei Medical University were enrolled in this retrospective observational study. Each participant received both CMT and mNGS during hospitalization. The inclusion criteria were as follows: age ≥18 years, the presence of at least one immunocompromised condition, a diagnosis of suspected pneumonia according to standard criteria, performance of bronchoalveolar lavage following a standard safety protocol, provision of BALF and other relevant samples (including blood and respiratory secretions) for both CMT and mNGS, and positive results for both CMT and mNGS (patients with negative pathogen tests were excluded).

Immunocompromised status in this study was defined as follows: active malignancies, excluding early-stage cancer or localized cutaneous malignancies; autoimmune diseases, including rheumatoid arthritis, connective tissue disease, systemic lupus erythematosus, and systemic vasculitis; solid tumors treated with chemotherapy; treatment with corticosteroids (prednisone, methylprednisolone, or dexamethasone); hematologic malignancies awaiting hematopoietic stem cell transplantation; and solid-organ transplantation, including renal and liver transplants.

Patients were diagnosed with suspected pneumonia if they exhibited new-onset chest imaging abnormalities (e.g., patchy/cloudy opacities or interstitial changes) along with at least one of the following: fever; new-onset cough or expectoration, with or without dyspnea; or a white blood cell count of >10 × 10^9^/L or <4 × 10^9^/L, with or without a left shift in neutrophils (Ramirez et al., 2020).

This study was approved by the Ethics Committee of the Second Hospital of Hebei Medical University. Because it was a retrospective study, written informed consent was not required.

### Data collection

Each patient underwent two types of pathogen testing—CMT and mNGS—during hospitalization. Baseline demographic characteristics were collected, including age, sex, intensive care unit (ICU) admission, development of severe pneumonia, presence of respiratory failure and ventilation use, pleural effusions, and occurrence of multiple organ dysfunction syndrome. CMT and mNGS results, along with other demographic data, were extracted by two experienced experts. Four types of treatment changes were analyzed: adjustment of anti-pathogen therapy (antifungal, antituberculous, or antibiotic) and initiation of high-dose glucocorticoids based on mNGS findings. Patient outcomes were categorized into four groups: improved, deteriorated, died, or discharged against medical advice.

### CMT

Three types of specimens—BALF, lower respiratory secretions, and blood serum—were used to perform CMT testing in all enrolled patients. BALF and lower respiratory secretions were cultured to detect bacterial and fungal pathogens. Blood serum and throat swab samples were used for real-time PCR to detect viral pathogens, including human herpesvirus, influenza A/B virus, and severe acute respiratory syndrome coronavirus 2 (SARS-CoV-2). Additionally, BALF and lower respiratory secretions were tested using the Xpert assay to detect *Mycobacterium tuberculosis*. Blood serum was also analyzed using the (1,3)-β-D-glucan test (G test) and the Gomori methenamine silver stain (GM test) for further detection of fungal pathogens.

### mNGS tests

#### Body fluid: sample processing and DNA extraction

A 1.5- to 3.0-mL BALF sample was collected from each patient according to standard procedures. A 1.5-mL microcentrifuge tube containing 0.6 mL of sample, enzyme, and 1 g of 0.5-mm glass beads was mounted on a horizontal platform vortex mixer and agitated vigorously at 2,800–3,200 rpm for 30 min. Then, 0.3 mL of the sample was transferred into a new 1.5-mL microcentrifuge tube, and DNA was extracted using the TIANamp Micro DNA Kit (DP316; TIANGEN BIOTECH) following the manufacturer’s recommendations (Long et al., 2016).

#### Construction of DNA libraries

DNA libraries were constructed through DNA fragmentation, end repair, adapter ligation, and PCR amplification. Library quality was assessed using the Agilent 2100 system. Qualified libraries were sequenced using the BGISEQ-50 or MGISEQ-2000 platform (Jeon et al., 2014).

#### Sequencing and bioinformatic analysis

High-quality sequencing data were generated by removing low-quality reads, followed by computational subtraction of human host sequences mapped to the human reference genome (hg19) using Burrows–Wheeler alignment. After removing low-complexity reads, the remaining data were classified by simultaneously aligning them to four microbial genome databases consisting of bacteria, fungi, viruses, and parasites. Classification reference databases were downloaded from NCBI (ftp://ftp.ncbi.nlm.nih.gov/genomes/). RefSeq includes 4,945 whole-genome sequences of viral taxa, 6,350 bacterial genomes or scaffolds, 1,064 fungal genomes related to human infection, and 234 parasite genomes associated with human disease (Li and Durbin, 2009).

### Clinical composite diagnosis as the reference standard

The patient’s medical records—including clinical features, laboratory examinations, microbiological tests (mNGS and CMT), chest imaging, and therapeutic responses—were independently reviewed by two physicians specializing in the management of infections in immunocompromised hosts. They determined whether the patients had an infectious etiology and identified the pathogens (definite or probable). When disagreement arose, an in-depth discussion was conducted, and a third senior physician was consulted if a consensus could not be reached.

### Statistical analysis

Continuous variables are reported as mean ± standard deviation or median (25th, 75th percentiles), depending on whether they followed a normal or non-normal distribution. Categorical variables are presented as numbers (percentages). Microbiological etiology and clinical composite diagnosis were used as reference standards, in accordance with previous studies (Cebular et al., 2003; Letourneau et al., 2014). The McNemar test was applied to compare the diagnostic performance of CMT and mNGS. Test concordance was assessed using the Kappa (κ) statistic. Sensitivity, specificity, and accuracy for each pathogen were calculated using standard formulas for proportions. Corresponding 95% confidence intervals were determined using Wilson’s method. All statistical tests were two-tailed, and a *p*-value of < 0.05 was considered statistically significant.

## Results

### Patient characteristics

In total, 146 patients were included in this study. Their mean age was 59.7 ± 14.7 years. Six immunocompromised statuses were analyzed: hematologic malignancy, autoimmune disease, solid tumor receiving chemotherapy, corticosteroid therapy, hematologic malignancy (listed twice in the original—see note below), and solid-organ transplantation. Among the 146 patients, 34 were admitted to the ICU, and 48 had severe pneumonia. Nineteen patients developed respiratory failure, including 18 cases of type I and 1 case of type II respiratory failure. Thirteen patients had pleural effusions, and four developed multiple organ dysfunction syndrome. During hospitalization, 19 patients received noninvasive ventilation, and 11 received invasive ventilation. A total of 72 patients underwent changes in anti-pathogen treatment based on the mNGS results, including shifts to antifungal, antituberculous, antiviral, or updated antibiotic therapies, as well as initiation of high-dose glucocorticoids (**Table 1**).

**Table 1:** Characteristics of immunocompromised CAP.

Characteristics	Clinical value
Age (year), mean ± SD	59.7 ± 14.7
Gender, male, *n* (%)	81 (55.5)
Immunocompromised status, *n* (%)	
Hematologic malignancy	2 (1.4)
Autoimmune disease	92 (63.0)
Solid tumor receiving chemotherapy	27 (18.5)
Receiving corticosteroid therapy	13 (8.9)
Hematologic malignancy	7 (4.8)
Solid-organ transplantation	5 (3.4)
Admitted to ICU	34 (23.3)
Severe pneumonia	48 (32.9)
Respiratory failure	19 (13.0)
Type I respiratory failure	18 (12.3)
Type II respiratory failure	1 (0.7)
Effusions	13 (8.9)
MODS	4 (2.7)
Using noninvasive ventilation	19 (13.0)
Using invasive ventilation	11 (7.5)
Change treatment refer to mNGS	72 (49.3)
Antifungus	30 (20.5)
Antituberculosis	3 (2.1)
Antivirus	13 (8.9)
Update antibiotics	15 (10.3)
Using high-dose glucocorticoid	4 (2.7)
Outcomes of all subjects	146 (100)
Improved	87 (59.6)
Deteriorate	3 (2.1)
Died	13 (8.9)
Against advice discharge	43 (29.5)
Outcomes of subjects after changing treatment refer to mNGS	72 (49.3)
Improved	45 (62.5)
Deteriorate	1 (1.38)
Died	3 (4.16)
Against advice discharge	23 (31.9)

CAP, community-acquired pneumonia; ICU, intensive care unit; MODS, multiple organ dysfunction syndrome; mNGS, metagenomic next-generation sequencing.

### Outcomes

**Figure 1:**
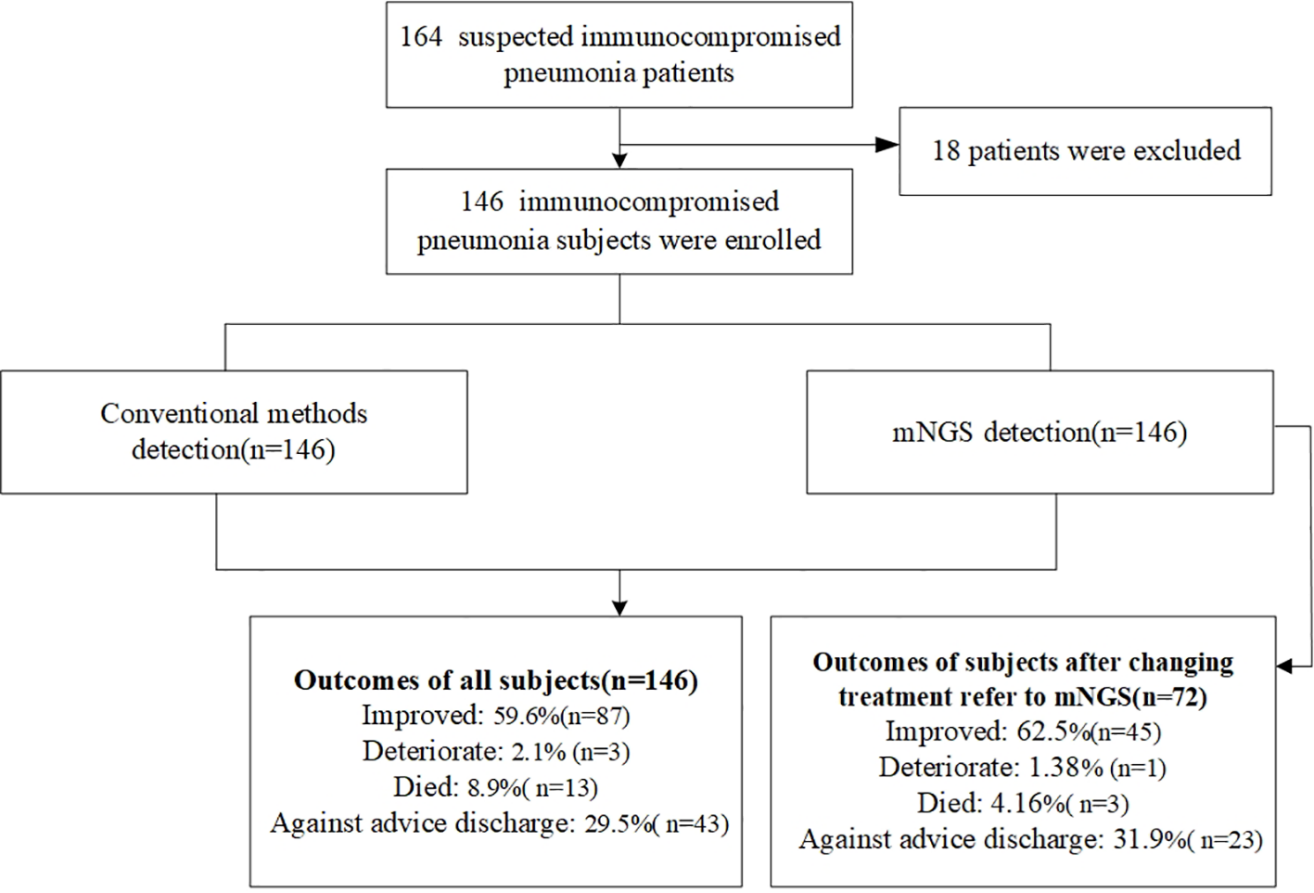
Study flowchart.

Among all patients, 87 showed clinical improvement. Three patients experienced deterioration, 13 died, and 43 were discharged against medical advice. Among the 72 patients whose treatment was adjusted based on mNGS results, 45 improved, 1 deteriorated, 3 died, and 23 were discharged against medical advice (**Table 1**, **Figure 1**).

### Pathogen detection by mNGS and CMT

**Figure 2:**
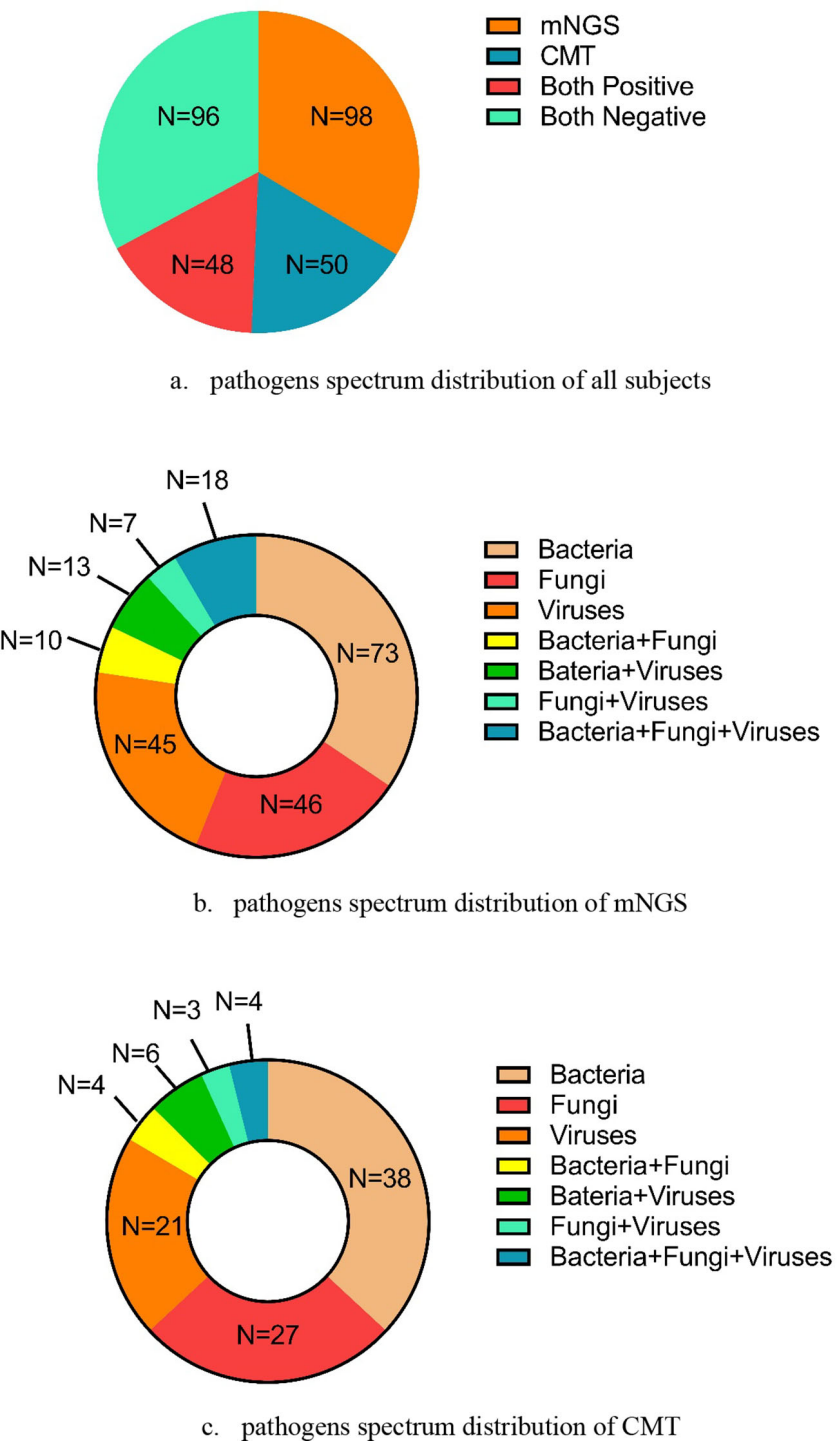
The pathogens’ spectrum by mNGS and CMT in all subjects. **(a)** Pathogens’ spectrum distribution of all subjects. **(b)** Pathogens’ spectrum distribution of mNGS. **(c)** Pathogens’ spectrum distribution of CMT.

As shown in **Figure 2**, 98 cases tested positive by mNGS, and 50 cases tested positive by CMT. Among these, 48 cases tested positive by both methods. The pathogen spectrum revealed that mNGS detected 73 bacterial, 46 fungal, and 45 viral pathogens, while CMT identified 38 bacterial, 27 fungal, and 21 viral pathogens (**Figure 2**). **Table 2** compares the number of positive detections between mNGS and CMT. For single-pathogen infections, the viral detection rate showed significant inconsistency between the two methods (*p* < 0.001, κ = 0.510). In mixed infections, cases involving bacteria and viruses also demonstrated significant inconsistency (*p* < 0.001, κ = 0.387).

**Table 2:** Pathogens in mNGS and CMT in all subjects.

Pathogens	mNGS	CMT	*p*	κ
Bacteria	73	38	0.451	0.055
Fungi	46	27	0.637	0.038
Viruses	45	21	<0.001	0.510
Bacteria + Fungi	10	4	0.582	−0.041
Bacteria + Viruses	13	6	<0.001	0.387
Fungi + Viruses	7	3	0.695	−0.030
Bacteria + Fungi + Viruses	6	4	0.006	0.239

mNGS, metagenomic next-generation sequencing; CMT, conventional microbiological testing.

### Pathogen spectrum of mNGS and CMT

**Figure 3:**
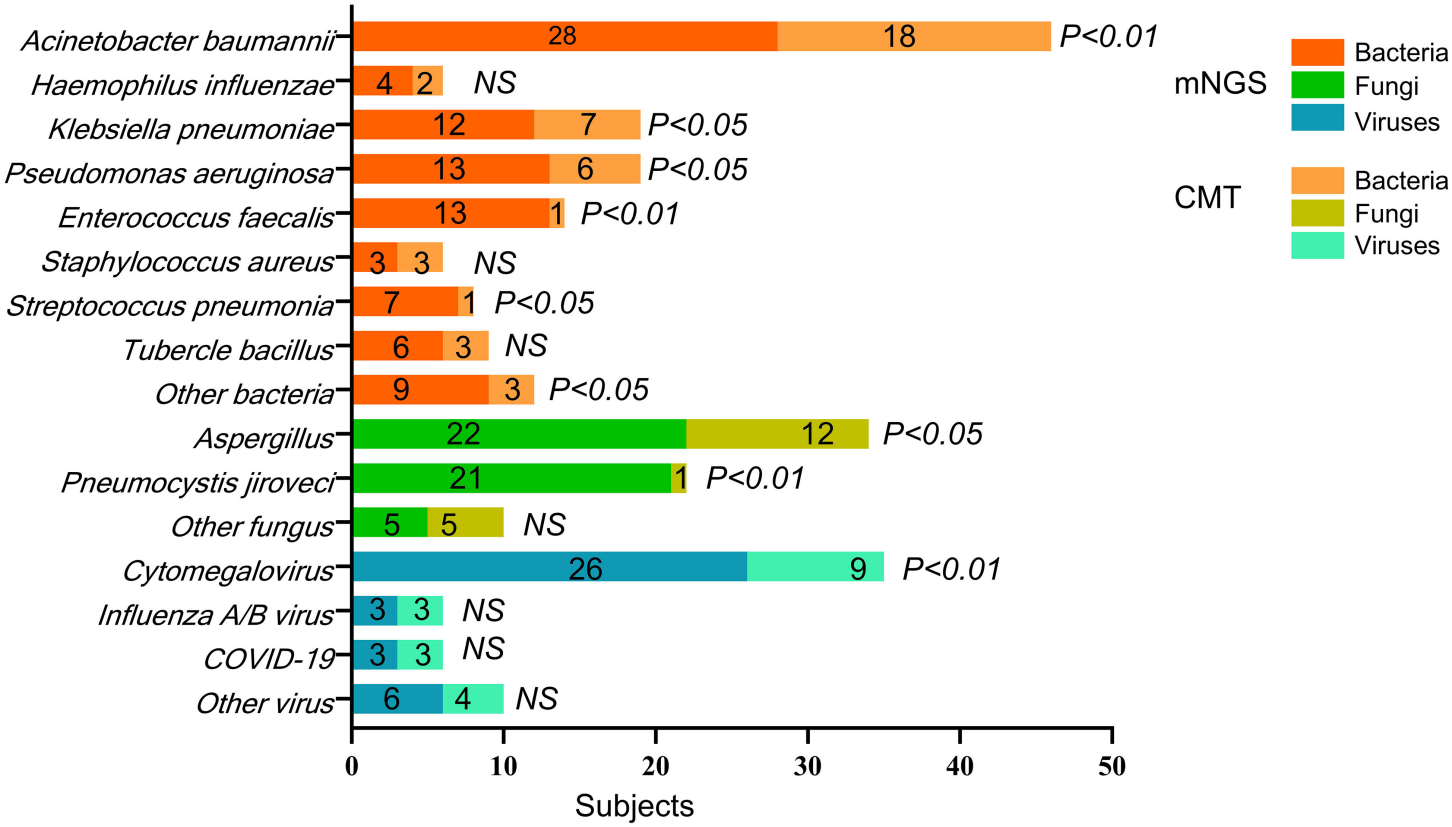
The pathogens’ spectrum of mNGS and CMT in all subjects. *p*-value refers to the comparison of mNGS and CMT test for the pathogen’s detection in all subjects. The specific *p*-value is shown in **Table 3**. NS, not significant.

**Figure 3** shows the distribution of pathogens identified in all patients using mNGS and CMT. Six colors were used to represent the pathogen spectrum detected by each method. In the bacterial spectrum, detection rates differed significantly between methods for several pathogens: *Acinetobacter baumannii* (28 vs. 18 cases, *p* < 0.001), *Klebsiella pneumoniae* (12 vs. 7 cases, *p* = 0.012), *Pseudomonas aeruginosa* (13 vs. 6 cases, *p* = 0.010), *Enterococcus faecalis* (13 vs. 1 case, *p* = 0.001), and a group of other bacteria including *Stenotrophomonas maltophilia*, *Corynebacterium striatum*, *Legionella pneumophila*, and *Nocardia farcinica* (9 vs. 3 cases, *p* = 0.016).

In the fungal spectrum, significant differences were observed in the number of positive cases for *Aspergillus* (22 vs. 12 cases, *p* = 0.014) and *Pneumocystis jirovecii* (21 vs. 1 case, *p* < 0.001). For *Candida albicans*, *Candida glabrata*, and *Candida tropicalis*, both mNGS and CMT detected four cases each, with no significant difference overall (5 vs. 5 cases, *p* = 0.158).

Finally, in the viral spectrum, cytomegalovirus was the most commonly detected virus by both mNGS (26 cases) and CMT (9 cases), with a significant difference in detection rates (*p* < 0.001). Both methods detected three cases each of influenza A/B virus and SARS-CoV-2. Additional viruses detected by mNGS included two cases of human herpesvirus 1, one case of Epstein–Barr virus, one case of human coronavirus NL63, and two cases of torque teno virus. CMT detected one case of human herpesvirus 1, one case of human coronavirus NL63, and two cases of torque teno virus.

### Pathogen spectrum detection by mNGS and CMT

**Table 3** summarizes the detection rates and consistency of pathogen spectra identified by mNGS and CMT across all patients. For bacterial pathogens, significant differences in detection rates between mNGS and CMT were observed for five species: *A. baumannii* (*p* < 0.001), *K. pneumoniae* (*p* = 0.012), *P. aeruginosa* (*p* = 0.010), *E. faecalis* (*p* = 0.001), and *Staphylococcus aureus* (*p* < 0.001). *S. aureus* showed high concordance between methods (κ = 0.710), while *A. baumannii* showed moderate concordance (κ = 0.485).

**Table 3:** The pathogens’ spectrum of mNGS and CMT in all subjects.

Pathogen Type	Specific Pathogen	All subjects				Subgroup subjects							
						Admitted to ICU				Combined severe pneumonia			
		mNGS (Yes, %)	CMT (Yes, %)	*p*	Kappa	mNGS (Yes, %)	CMT (Yes, %)	*p*	Kappa	mNGS (Yes, %)	CMT (Yes, %)	*p*	Kappa
Bacterial	*Acinetobacter baumannii*	28	18	<0.001	0.485	9	9	0.201	0.175	13	8	0.014	0.409
	*Haemophilus influenzae*	4	2	0.068	0.271	1	0	–	–	2	0	–	–
	*Klebsiella pneumoniae*	12	7	0.012	0.272	3	4	0.225	0.182	5	3	0.001	0.412
	*Pseudomonas aeruginosa*	13	6	0.010	0.275	2	4	0.522	-0.090	4	6	0.002	0.397
	*Enterococcus faecalis*	13	1	0.001	0.132	4	1	0.001	0.389	9	1	0.035	0.340
	*Staphylococcus aureus*	3	3	0.835	0.710	3	2	<0.001	0.785	3	3	<0.001	0.789
	*Streptococcus pneumoniae*	7	1	0.012	−0.012	1	0	–	–	1	0	–	–
	Tubercle bacillus	6	3	0.717	−0.028	0	0	–	–	0	0	–	–
	Other bacteria	9	3	0.016	−0.032	2	2	0.716	−0.032	5	3	0.542	−0.085
Fungus Virus	*Aspergillus*	22	12	0.014	0.408	4	3	0.812	0.341	10	8	0.759	0.355
	*Pneumocystis jirovecii*	21	1	<0.001	0.479	9	0	–	–	11	1	0.006	0.492
	Other fungi	5	5	0.158	0.152	0	1	–	–	2	2	<0.001	0.478
	Cytomegalovirus	26	9	<0.001	0.465	6	2	0.002	0.452	17	7	<0.001	0.475
	Influenza A/B virus	3	3	0.857	1	1	1	0.834	1	2	2	0.827	1
	COVID-19	3	3	0.932	1	2	2	0.915	1	7	7	0.910	1
	Other virus	6	4	0.569	−0.035	2	0	–	–	4	5	0.590	−0.077

mNGS, metagenomic next-generation sequencing; CMT, conventional microbiological testing. Other bacteria refer to *Stenotrophomonas maltophilia*, *Corynebacterium striatum*, *Legionella pneumophila*, and *Nocardia farcinica*. Other fungi refer to *Candida albicans*, *Candida glabrata*, and *Candida tropicalis.* Other viruses refer to human herpesvirus 1, Epstein–Barr virus, human coronavirus NL63, and torque teno virus.

For fungal pathogens, detection rates differed significantly for *Aspergillus* (*p* = 0.014) and *P. jirovecii* (*p* < 0.001). None of the fungal species demonstrated strong detection consistency between mNGS and CMT.

For viral pathogens, a significant difference in detection rates was observed for cytomegalovirus (*p* < 0.001), whereas no significant differences were found for influenza A/B virus (*p* = 0.857) or SARS-CoV-2 (*p* = 0.932). Both influenza A/B virus and SARS-CoV-2 exhibited perfect concordance between the two methods (κ = 1).

### Subgroup analysis

**Table 3** presents a subgroup analysis of patients who were either admitted to the ICU or had severe pneumonia. Among ICU patients, the detection rates of two bacterial pathogens—*E. faecalis* (*p* = 0.001) and *S. aureus* (*p* < 0.001)—were significantly different between mNGS and CMT. In patients with severe pneumonia, significant differences were observed for four bacterial pathogens: *K. pneumoniae* (*p* = 0.001), *P. aeruginosa* (*p* = 0.002), *E. faecalis* (*p* = 0.035), and *S. aureus* (*p* < 0.001). Notably, *S. aureus* was the only bacterial pathogen that showed high concordance between mNGS and CMT in both subgroups—ICU patients (κ = 0.785) and patients with severe pneumonia (κ = 0.789).

In patients with severe pneumonia, detection of *P. jirovecii* also differed significantly between the two methods (*p* = 0.006). None of the fungal pathogens showed strong detection consistency between mNGS and CMT in either ICU patients or those with severe pneumonia. For viral pathogens, cytomegalovirus detection rates differed significantly between mNGS and CMT in both subgroups (*p* = 0.002 for ICU patients, *p* < 0.001 for those with severe pneumonia). However, no significant differences were observed for influenza A/B virus (*p* = 0.834 and *p* = 0.827, respectively) or SARS-CoV-2 (*p* = 0.915 and *p* = 0.910, respectively). Both influenza A/B virus and SARS-CoV-2 showed perfect agreement between mNGS and CMT (κ = 1) in ICU patients and those with severe pneumonia.

### Diagnostic performance and accuracy of CMT and mNGS

**Tables 4** and **5** show the diagnostic performance and accuracy of CMT and mNGS in all patients. For bacterial detection, the diagnostic accuracy of *A. baumannii* detection was significantly higher in the mNGS group than in the CMT group (79.1% vs. 66.3%, *p* = 0.032) (**Table 5**). In *Haemophilus influenzae* detection, mNGS showed higher sensitivity (75.0% vs. 25.0%) and positive predictive value (PPV) (80.0% vs. 50.0%) than CMT (**Table 4**). For *K. pneumoniae*, both mNGS and CMT demonstrated 100% specificity and PPV, suggesting that both methods were reliable for detecting this pathogen (**Table 4**). Nevertheless, mNGS yielded higher diagnostic accuracy than CMT for both *H. influenzae* (85.0% vs. 71.0%, *p* = 0.029) and *K. pneumoniae* (85.0% vs. 71.0%, *p* = 0.040) (**Table 4**). In *P. aeruginosa* detection, the specificity of mNGS and CMT was similar. However, mNGS demonstrated higher PPV (92.8% vs. 85.7%) and diagnostic accuracy (92.5% vs. 67.5%, *p* = 0.006) than CMT (**Tables 4**, **5**).

**Table 4:** Diagnostic performance of CMTs and mNGS in all subjects.

Pathogen Type	Specific Pathogen	mNGS				CMT			
		Sensitivity% (95% CI)	Specificity% (95% CI)	PPV% (95% CI)	NPV% (95% CI)	Sensitivity% (95% CI)	Specificity% (95% CI)	PPV% (95% CI)	NPV% (95% CI)
Bacterial pneumonia	*Acinetobacter baumannii*	70.6 (63.2–77.9)	87.6 (82.2–92.9)	62.2 (54.3–70.0)	90.7 (85.9–96.4)	41.2 (33.2–49.1)	91.5 (86.9–96.0)	60.0 (52.1–67.9)	83.1 (77.0–89.2)
	*Haemophilus influenzae*	75.0 (82.0–67.9)	99.2 (97.7–100.0)	80.0 (86.4–73.5)	99.0 (97.4–100.0)	25.0 (17.9–32.0)	99.3 (97.9–100.0)	50.0 (41.9–58.1)	99.0 (97.4–100.0)
	*Klebsiella pneumoniae*	71.4 (64.1–78.7)	100.0 (100.0–100.0)	100.0 (100.0–100.0)	96.4 (93.4–99.4)	42.9 (34.9–50.9)	100.0 (100.0–100.0)	100(100.0–100.0)	93.9 (90.0–97.9)
	*Pseudomonas aeruginosa*	85.7 (80.0–91.4)	99.2 (97.7–100.0)	92.8 (88.6–96.9)	98.5 (96.5–100.0)	35.7 (27.9–43.5)	99.2 (97.7–100.0)	85.7 (80.0–91.4)	92.7 (88.5–96.9)
	*Enterococcus faecalis*	83.3 (77.2–89.3)	94.3 (90.5–98.1)	61.9 (54.0–69.7)	97.7 (95.3–100.0)	0.0 (0–0)	99.3 (97.9–100.0)	50.0 (41.9–58.1)	0.0 (0–0)
	*Staphylococcus aureus*	50.0 (41.3–58.1)	99.3 (97.9–100.0)	75.0 (67.9–82.0)	97.9 (95.6–100.0)	75.0 (68.0–82.0)	100.0 (100.0–100.0)	100.0 (100.0–100.0)	99.3 (97.9–100.0)
	*Streptococcus pneumoniae*	100.0 (100.0–100.0)	99.3 (97.9–100)	87.5 (82.1–92.8)	100.0 (100.0–100.0)	0.0 (0–0)	99.3 (97.9–100)	50.0 (41.3–58.1)	100 (100.0–100.0)
	Tubercle bacillus	71.4 (64.1–78.7)	99.3 (97.9–100)	87.5 (82.1–92.8)	100.0 (100.0–100.0)	28.6 (21.3–35.9)	99.3 (97.9–100)	75.0 (68.0–82.0)	94.7 (91.1–98.3)
Fungus	*Aspergillus*	81.3 (75.0–87.6)	93.1 (88.9–97.2)	70.9 (63.5–78.3)	96.1 (93.0–99.2)	43.8 (35.7–51.8)	96.2 (93.1–99.3)	70.5 (63.1–77.9)	89.9 (85.0–94.8)
	*Pneumocystis jirovecii*	14.3 (8.62–20.0)	100.0 (100.0–100.0)	100.0 (100.0–100.0)	49.8 (41.6–57.9)	100.0 (100.0–100.0)	89.9 (85.0–94.8)	90.1 (85.3–94.9)	100 (100.0–100.0)
Virus	Cytomegalovirus	100.0 (100.0–100.0)	87.6 (82.3–92.9)	60.5 (52.6–68.4)	100 (100.0–100.0)	66.7 (59.1–74.3)	97.8 (95.4–100)	75.0 (68.0–82.0)	96.5 (93.5–99.4)
	Influenza A/B virus	100.0 (100.0–100.0)	100.0 (100.0–100.0)	100.0 (100.0–100.0)	100.0 (100.0–100.0)	100.0 (100.0–100.0)	100.0 (100.0–100.0)	100.0 (100.0–100.0)	100.0 (100.0–100.0)
	COVID-19	100.0 (100.0–100.0)	98.6 (96.7–100.0)	60.0 (52.0–67.9)	100.0 (100.0–100.0)	100.0 (100.0–100.0)	98.6 (96.7–100.0)	60.0 (52.0–67.9)	100.0 (100.0–100.0)

mNGS, metagenomic next-generation sequencing; CMT, conventional microbiological testing; PPV, positive predictive value; NPV, negative predictive value.

**Table 5:** Diagnostic accuracy of CMTs and mNGS in all subjects.

Pathogen Type	Specific Pathogen	mNGS	CMT	*p*
Bacterial pneumonia	*Acinetobacter baumannii*	79.1 (65.9–92.3)	66.3 (50.7–82.0)	0.032
	*Haemophilus influenzae*	86.8 (61.3–100.0)	62.1 (49.6–94.7)	0.029
	*Klebsiella pneumoniae*	85.0 (70.6–99.4)	71.0 (53.5–88.6)	0.040
	*Pseudomonas aeruginosa*	92.5 (81.6–100.0)	67.5 (49.7–85.2)	0.006
	*Enterococcus faecalis*	88.8 (71.2–100.0)	50.4 (36.8–73.9)	0.025
	*Staphylococcus aureus*	75.2 (55.1–98.0)	87.8 (62.7–100.0)	0.093
	*Streptococcus pneumoniae*	99.6 (96.0–100.0)	50.4 (36.8–73.9)	0.004
	Tubercle bacillus	85.4 (65.0–100.0)	63.9 (39.1–88.8)	0.014
Fungus	*Aspergillus*	77.2 (65.7–93.7)	70.0 (53.7–86.2)	0.639
	*Pneumocystis jirovecii*	95.0 (91.2–98.7)	57.1 (33.1–81.2)	0.011
Virus	Cytomegalovirus	93.8 (89.7–97.9)	82.2 (63.4–100.0)	0.045
	Influenza A/B virus	100 (100–100)	100 (100–100)	–
	COVID-19	99.3 (98.1–100.0)	99.3 (98.1–100.0)	–

mNGS, metagenomic next-generation sequencing; CMT, conventional microbiological testing.

In this study, CMT showed 0.0% sensitivity and 50.0% PPV for both *E. faecalis* and *Streptococcus pneumoniae*, indicating poor detection ability for these pathogens using conventional methods (**Table 4**). In contrast, mNGS showed measurable sensitivity and PPV for both, and its diagnostic accuracy was significantly higher than that of CMT: 88.8% vs. 50.4% (*p* = 0.025) for *E. faecalis* and 99.6% vs. 50.4% (*p* = 0.004) for *S. pneumoniae* (**Table 5**). Additionally, CMT showed higher sensitivity (75.0% vs. 50.0%) and PPV (100.0% vs. 75.0%) for *S. aureus* (**Table 4**), but the difference in diagnostic accuracy between mNGS and CMT was not statistically significant (75.2% vs. 87.3%, *p* = 0.093) (**Table 5**). For *M. tuberculosis*, the methods showed identical specificity (99.3%). However, mNGS yielded significantly higher sensitivity, PPV, and diagnostic accuracy than CMT (**Tables 4**, **5**).

Two fungal pathogens were analyzed: *Aspergillus* and *P. jirovecii*. mNGS demonstrated higher sensitivity in detecting *Aspergillus* than CMT (81.3% vs. 43.8%) (**Table 4**). The diagnostic accuracy of mNGS was also significantly higher than that of CMT in detecting *P. jirovecii* (95.0% vs. 57.1%, *p* = 0.011) (**Table 5**). However, for this pathogen, CMT showed 100% sensitivity, while mNGS only achieved 14.3%. The overall difference in diagnostic accuracy between mNGS and CMT for *P. jirovecii* was not statistically significant (77.2% vs. 70.0%, *p* = 0.639) (**Table 5**).

For viruses, diagnostic performance and accuracy for influenza A/B virus and SARS-CoV-2 were highly consistent between mNGS and CMT. The diagnostic PPV and accuracy for influenza A/B virus were both 100% with both methods (**Tables 4**, **5**). Similarly, the diagnostic accuracy for SARS-CoV-2 was 99.3% for both mNGS and CMT (**Table 5**). In contrast, for cytomegalovirus detection, mNGS demonstrated lower specificity (87.6% vs. 97.8%) but higher sensitivity (100.0% vs. 66.7%) than CMT (**Table 4**). Moreover, mNGS showed significantly higher diagnostic accuracy for cytomegalovirus (93.8% vs. 82.2%, *p* = 0.045) (**Table 5**).

**Supplementary File 1. Table 1** shows the diagnostic performance of single and mixed infections in all patients. For single bacteria and fungi detection, the diagnostic specificity of both the mNGS and the CMT group was higher than 90% (bacteria, 94.3% vs. 90.3%; fungi, 90.5% vs. 93.5%), suggesting the ability of these two methods in correctly identifying individuals with non-single bacteria or fungi infection. For single virus detection, the sensitivity and negative predictive value (NPV)% were both 100% in the mNGS group, which indicated that the detection capability of mNGS is superior compared to CMT. However, the specificity (96.7% vs. 89.6%) and NPV% (93.2% vs. 89.7%) of bacteria combined with fungi infection were higher in CMT compared to mNGS. In bacteria combined with virus infection, the diagnostic specificity of CMT was also higher than that of mNGS (99.3% vs. 90.3%). Moreover, the specificity (93.5% vs. 95.0%) and NPV% (90.0% vs. 94.3%) of fungi combined with virus infection were both higher than 90% in the two methods, demonstrating the ability of both methods in identifying non-fungi combined with virus-infected individuals. Finally, in bacteria combined with fungi and virus infection, the specificity (97.5% vs. 90.3%) and NPV% (93.8% vs. 88.7%) of CMT were higher compared to mNGS (**Supplementary File 1. Table 1.**).

## Discussion

Because of the rapid systemic spread and high lethality, the diagnosis of CAP is more complex in immunocompromised patients than in the general population. Immunocompromised patients are particularly vulnerable to severe infections caused by Gram-negative bacilli (Cebular et al., 2003; Letourneau et al., 2014). Furthermore, patients undergoing post-transplant immunosuppression, those with hematologic malignancies, or those receiving immunosuppressive therapies face elevated risks of *P. jirovecii* and *Aspergillus* infections. These pathogens represent opportunistic organisms that frequently lead to severe pneumonia in this population. The prolonged culture time, stringent culture requirements, and limited diagnostic reliability of serological testing have restricted the application of CMT in immunocompromised pneumonia.

BALF refers to a diagnostic method for analyzing pathogens by obtaining BALF from the site of airway infection (Connett, 2000). The mNGS can be characterized by unbiasedness, deep sequencing, and direct nucleic acid detection; this technique does not rely on host immune status so that it can effectively analyze pathogens in immunocompromised patients with pneumonia (Han et al., 2019; Li et al., 2022). The mNGS-BALF approach delivers results within 24–48 h, thus effectively enhancing diagnostic timeliness. Therefore, the mNGS-BALF approach enables earlier targeted therapy, facilitating more timely clinical interventions (Han et al., 2019; Li et al., 2022).

In this study, the diagnostic performance analysis suggests that both mNGS and CMT have their own strengths in detecting specific bacterial and fungal pathogens, while their performance and accuracy in viral pathogen detection were similar. Moreover, compared with CMT, mNGS demonstrated significantly higher detection rates for one bacterial pathogen (*E. faecalis*) and one fungal pathogen (*P. jirovecii*) in immunocompromised patients who were admitted to the ICU or had severe pneumonia. In addition, the condition of 45 patients improved after treatment adjustments based on mNGS results, suggesting that the clinical utility of mNGS is well supported.

In this study, the pathogen spectrum showed that more total positive samples were detected using mNGS (*n* = 98) than using CMT (*n* = 50) across all three microbe types. In the three single-pathogen infection modes (bacterial-only, fungal-only, and viral-only infections) and four mixed infection modes (bacterial + fungal, bacterial + viral, fungal + viral, and bacterial + fungal + viral infections), mNGS also yielded more positive results than CMT (**Figure 2**). Among these infection patterns, bacterial and fungal detections showed low concordance between the two methods, while viral detection demonstrated moderate agreement (κ = 0.510, *p* < 0.001). Although previous studies have reported the diagnostic performance of mNGS in the general population, evidence specific to BALF-based mNGS in immunocompromised patients with pneumonia remains limited. Sun et al (Sun et al., 2024). analyzed 69 immunocompromised Chinese patients with suspected pneumonia and found significant differences between mNGS and CMT in bacterial and fungal detection (*p* < 0.05 for both), although the difference in viral detection was not statistically significant (*p* > 0.05). In contrast, Peng et al (Peng et al., 2021). reported similar detection performance for bacteria, fungi, and viruses between mNGS and CMT. Their study involved immunocompromised patients with more combined underlying conditions and a smaller sample size, which may account for the differing results compared with our study.

The pathogen detection spectrum of mNGS and CMT showed that detection rates varied notably between the two methods for different pathogens. Among bacterial pathogens, *A. baumannii*—a highly antibiotic-resistant organism known to cause severe and often fatal pneumonia infections (Mea et al., 2021)—was detected at a significantly higher rate by mNGS than by CMT in immunocompromised patients. This suggests that mNGS has greater sensitivity for identifying this nosocomial pathogen. Similarly, Wang et al (Wang et al., 2024). analyzed BALF samples from 112 patients with pneumonia in a tertiary hospital in China and found that the detection rate of *A. baumannii* using mNGS was significantly higher than with CMT.

Although patient populations may differ, significant benefits of mNGS have been demonstrated in detecting this common multidrug-resistant bacterium. Notably, the detection rate and consistency for *S. aureus* were similar between mNGS and CMT. *S. aureus* is a common Gram-positive pathogen with high virulence, capable of causing rapidly life-threatening infections by invading the skin, soft tissues, and internal organs (Chua et al., 2014; Kwiecinski and Horswill, 2020). In this study, both methods showed comparable detection rates and high concordance, suggesting that the sensitivity and specificity of mNGS and CMT are both reliable for identifying this pathogen. On the one hand, mNGS can detect potential pathogens more rapidly than CMT, allowing earlier adjustments to clinical management. On the other hand, CMT is more cost-effective and relies on more accessible specimen processing. Therefore, both mNGS and CMT may serve as valuable tools for the detection of *S. aureus*.

In patients with pneumonia, ICU admission and severe pneumonia are both associated with poor outcomes, including disease deterioration, a poor prognosis, and an increased mortality risk. In this study, 34 ICU-admitted patients and 48 with severe pneumonia were analyzed in subgroup comparisons. In both groups, mNGS showed significantly higher detection rates for *E. faecalis* and *P. jirovecii* than CMT. *E. faecalis* is a Gram-positive facultative anaerobe and ranks among the most prevalent microorganisms associated with severe hospital-acquired infections. In both the ICU and severe pneumonia subgroups, mNGS demonstrated a significantly higher detection rate for *E. faecalis* than CMT, suggesting superior sensitivity in critically ill patients. Supporting this, a case report highlighted the utility of mNGS in identifying *E. faecalis* from a urine sample in a culture-negative, severe urinary tract infection case, leading to effective targeted therapy. This underscores the potential of mNGS to serve as a supplementary diagnostic tool for severe infections across different systems when conventional methods fail (Li et al., 2020). *P. jirovecii*, a common cause of opportunistic lung infections, is associated with high mortality in immunocompromised individuals (Salzer et al., 2018). Because of their weakened immune defenses, these patients are particularly susceptible to severe *P. jirovecii* pneumonia (PCP), often requiring intensive care (Salzer et al., 2018; Giacobbe et al., 2023). Both the primary and subgroup analyses in this study indicated that mNGS had a significantly higher detection rate for PCP than CMT, suggesting superior efficiency in diagnosing this condition. In 2022, Wang et al. conducted a retrospective study evaluating mNGS for PCP diagnosis, and their analysis of 122 immunosuppressed patients similarly confirmed that mNGS outperformed traditional methods—including the GM and G tests—for detecting fungal pathogens (Wang et al., 2022).

In this study, the sensitivity, specificity, PPV, and NPV were used to evaluate the diagnostic performance of mNGS and CMT. The PPV represents a method’s ability to correctly identify true-positive samples and serves as a useful indicator of diagnostic value. Our analysis showed that mNGS yielded higher PPVs than CMT for seven bacterial pathogens, with the exception of *S. aureus*, suggesting that each method has its strengths in detecting specific bacterial species. Although both methods showed the same specificity, mNGS demonstrated higher sensitivity and PPV overall, indicating superior diagnostic performance. Li et al (Li et al., 2023). conducted a meta-analysis of 18 studies involving BALF pathogens in patients with pneumonia and concluded that mNGS exhibits high sensitivity and strong diagnostic capacity, particularly in critically ill or immunocompromised individuals. More recently, Chen et al. analyzed 266 patients with suspected pneumonia in southern China to compare mNGS and CMT, finding that mNGS demonstrated diagnostic advantages in tuberculosis (80.0% sensitivity and 97.4% specificity) (Chen et al., 2022). In line with these findings, our study supports the consistent diagnostic value of mNGS for various bacterial pathogens, reinforcing its potential for broad clinical application.

Regarding fungal pathogens, both mNGS and CMT had PPVs above 90%, although their sensitivities differed. CMT showed higher sensitivity for *Aspergillus* detection, while mNGS was more sensitive in detecting *P. jirovecii*. Despite this inconsistency in sensitivity, mNGS demonstrated higher overall diagnostic accuracy for both fungal pathogens. In a meta-analysis of 418 patients with PCP, Li reported diagnostic accuracies of 0.987 in general and 0.985 in immunocompromised populations, affirming the utility of mNGS for diagnosing PCP (Li et al., 2023). *Aspergillus*, another invasive fungal pathogen, is a major cause of morbidity and mortality in immunocompromised patients. In our study, although mNGS exhibited higher sensitivity than CMT in detecting *Aspergillus*, its specificity was lower, and there was no significant difference in diagnostic accuracy between the two methods. This suggests that mNGS may not be consistently superior to CMT for diagnosing *Aspergillus* infections in this population. Jia et al. reported mNGS accuracy rates of 80.5% and 73.7% in immunocompetent and immunocompromised patients with pulmonary aspergillosis, respectively (Jia et al., 2023). We speculate that the patient’s immune status may influence the sensitivity and diagnostic accuracy of mNGS for *Aspergillus*. Given the complexity of underlying conditions in immunocompromised patients, further evidence is needed to better assess the diagnostic performance of mNGS for *Aspergillus* detection.

In this study, both mNGS and CMT showed high sensitivity, PPV, and diagnostic accuracy for influenza A/B virus and SARS-CoV-2, indicating consistent diagnostic performance between the two methods. Consistent with our findings, Bal et al. also reported a strong correlation between mNGS and reverse transcription PCR (RT-PCR) in detecting viral pathogens (Bal et al., 2018). The core process for CMT detection of influenza A/B virus and SARS-CoV-2 involves collecting oropharyngeal or nasopharyngeal swab specimens and analyzing them using RT-PCR. RT-PCR amplifies the target gene sequence to detect its presence in the original specimen. Similarly, mNGS relies on the extraction and sequencing of genetic material from the specimen, enabling detection through analysis of DNA fragments. Given the methodological similarities between mNGS and RT-PCR, we speculate that this underlies the comparable diagnostic performance of mNGS and CMT for these two viral pathogens.

Finally, we evaluated the clinical value of mNGS in improving patient prognosis. In this study, the conditions of 45 patients improved after treatment adjustments guided by mNGS results, suggesting that mNGS can aid in optimizing antibiotic therapy. Previous studies have also shown the advantages of mNGS using BALF samples in identifying the pathogenic etiology of pneumonia across various populations. Yang et al (Yang et al., 2022). collected BALF samples from 112 children with confirmed pneumonia and found that after adjusting antibiotic treatments based on mNGS and CMT results, 109 children (97.32%) showed clinical improvement. More recently, Hao et al (Hao et al., 2023). studied 266 patients with suspected pulmonary infections from southernmost China and recorded the clinical outcomes for each patient. More than half of the patients had their empirical treatments modified based on mNGS results, supporting the method’s potential to enhance pathogen identification and antimicrobial stewardship in clinical practice. These findings indicate the potential value of mNGS in guiding antibiotic treatment decisions across different patient populations. While evidence for its impact on outcomes in immunocompromised patients with pneumonia remains limited, this study provides support for mNGS as a promising diagnostic tool to improve prognosis in this high-risk group.

Although mNGS is a promising diagnostic technology, it is often limited in assisting clinicians with rapid clinical decisions because of the potential for overdiagnosis, high costs, and difficulties in interpreting results. First, lab contamination, skin and oral flora interference, and DNA from dead pathogens are three main factors that increase the risk of overdiagnosis, leading to false-positive results (Gu et al., 2019; Batool and Galloway-Peña, 2023; Diao et al., 2023). Li et al. have evaluated the performance of 122 mNGS laboratories and reported the source of the false-positive results in mNGS. They proved that the major cause of the false-positive results in mNGS is laboratory contamination, revealing that wet labs were the source of pollution in 68.56% of the cases (Diao et al., 2023). Therefore, strict adherence to both reagent and workflow quality control protocols is essential to minimize laboratory contamination. Flora interference is another factor that may increase the false-positive risk of mNGS. As humans host numerous commensal organisms, distinguishing true infection pathogens from skin or oral microbiota poses another challenge in interpreting mNGS results. For example, reads mapping to *Cutibacterium acnes* are commonly seen in mNGS libraries due to the contamination of the background sample or reagent (Gu et al., 2019). Using different threshold levels, setting sequencing reads to detect specific pathogens may effectively reduce flora interference (Batool and Galloway-Peña, 2023). In addition, lingering DNA from dead pathogens could be detected by mNGS, adding the risk of overdiagnosis. The detection of RNA is a potential solution whose abundance is directly correlated with the degree of gene transcription activity so that it can distinguish dead and live organisms in a clinical sample (d’Humières et al., 2021; Chen et al., 2022). Second, compared to CMT, mNGS incurs higher costs, resulting in a substantial proportion of out-of-pocket payments (Zhao et al., 2024). The high cost of mNGS remains a challenge, restricting its widespread application in clinical practice. Diao et al. reported an average cost of approximately 400 dollars per sample, exceeding that of any single traditional pathogen test in China (Diao et al., 2023). Further clinical studies and economic evaluations are needed to comprehensively evaluate the cost-effectiveness of mNGS in improving patient outcomes and clinical values (Chiu and Miller, 2019). Third, the interpretation of mNGS results is a complicated process. Bioinformatic biases emerging from different reference databases and taxonomic misclassifications may compromise pathogen identification from sequencing data, further complicating the interpretation of mNGS reports (Diao et al., 2021; López-Labrador et al., 2021). Setting standardized detection thresholds for detection platforms and adopting appropriate taxonomic tools and parameters based on clinical practice may help reduce the difficulty in interpreting reports. In summary, standardizing methodological protocols, bioinformatic pipelines, and reference databases is critical for the effective clinical implementation of mNGS diagnostics (Diao et al., 2021; Su et al., 2024).

This study has several limitations. First, multicenter prospective studies need to be designed and conducted. These studies would further support the findings and comprehensively evaluate the diagnostic value of mNGS for immunocompromised patients with pneumonia in real-world settings. In the future, we will further expand our multicenter research to investigate the diagnostic performance of mNGS in patients with diverse immunodeficiency types and co-infections. Second, developing and validating integrated clinical diagnostic algorithms can support tools that clarify optimal application scenarios, detection timing, and result interpretation criteria for mNGS in diagnosing immunocompromised pneumonia. This will assist clinicians in determining the appropriate timing for mNGS application and guide targeted therapeutic decisions. Third, it is important to optimize mNGS technology to improve specificity in challenging pathogen infections, such as complex fungal or opportunistic infections, while reducing costs and turnaround time to enhance its accessibility and cost-effectiveness in various clinical settings. Furthermore, establishing standardized mNGS testing procedures and interpretation guidelines will facilitate clinicians’ standardized reporting practices, enhancing diagnostic accuracy and clinical utility.

## Conclusions

Both mNGS and CMT have strengths in detecting specific bacterial and fungal pathogens in immunocompromised patients. Compared with CMT, mNGS demonstrated higher diagnostic performance and accuracy in detecting *E. faecalis* and *P. jirovecii*, particularly in patients admitted to the ICU and those with severe pneumonia. As a novel diagnostic tool, mNGS may significantly enhance pathogen detection and help improve clinical outcomes in immunocompromised populations.

## Data Availability

The data presented in the study are deposited in the "https://www.jianguoyun.com/p/DbbLU08QsZDVDRjPvIgGIAA" repository, accession number "hdSwEL".
