# Supplementary material for: Diagnostic value of metagenomic next generation sequencing of bronchoalveolar lavage fluid in immunocompromised patients with pneumonia
Source: Front Cell Infect Microbiol. 2025 Sep 16;15:1602636. doi: 10.3389/fcimb.2025.1602636 (PMC12479419; doi:10.3389/fcimb.2025.1602636)
Supplement: Supplementary file 1 [file Table1.docx]

**Supplementary file 1. Table 1. Diagnostic performance of CMTs and mNGS in single and mixed pathogen infections.**

| Methods | mNGS | | | | CMT | | | |
| --- | --- | --- | --- | --- | --- | --- | --- | --- |
| Pathogens | Sensitivity%  (95% CI) | Specificity%  (95% CI) | PPV%  (95% CI) | NPV%  (95% CI) | Sensitivity%  (95% CI) | Specificity%  (95% CI) | PPV%  (95% CI) | NPV%  (95% CI) |
| Bacteria | 73.5  (67.0-82.0) | 94.3  (90.5-98.1) | 89.5  (84.5-95.3) | 93.5  (89.2-99.4) | 42.8  (34.5-50.2) | 90.3  (84.9-93.4) | 72.0  (69.0-81.2) | 91.7  (87.4-96.0) |
| Fungi | 78.6  (70.7-85.4) | 90.5  (85.7-94.2) | 72.9  (65.7-80.1) | 93.1  (90.6-96.8) | 54.6  (46.9-62.8) | 93.5  (90.1-96.7) | 70.3  (62.9-77.1) | 90.2  (86.7-93.4) |
| Viruses | 100.0  (100.0-100.0) | 93.1  (88.4-98.6) | 70.4  (62.7-78.2) | 100.0  (100.0-100.0) | 74.8  (67.9-81.5) | 97.9  (94.6-100.0) | 72.3  (64.5-80.9) | 97.9  (94.3-100.0） |
| Bacteria+Fungi | 70.5  (63.2-76.8) | 89.6  (84.8-95.3) | 65.3  (59.2-71.8) | 89.7  (82.3-95.2) | 41.5  (33.2-49.8) | 96.7  (92.8-99.8) | 86.3  (81.5-92.6) | 93.2  (89.9-97.5) |
| Bateria+Viruses | 73.3  (67.2-79.3) | 90.3  (87.5-95.5) | 62.0  (54.5-70.1) | 92.7  (90.3-95.2) | 43.5  (35.7-52.8) | 99.3  (97.9-100.0) | 85.9  (80.3-92.6) | 91.5  (87.6-95.4) |
| Fungi+Viruses | 66.2  (58.3-74.9) | 93.5  (87.9-98.3) | 74.8  (66.3-83.9) | 90.0  (87.4-92.8) | 56.7  (49.8-63.2) | 95.0  (92.4-98.3) | 87.9  (81.3-94.0) | 94.3  (90.9-97.8) |
| Bacteria+Fungi+Viruses | 67.6  (59.5-76.3) | 90.3  (89.9-95.7) | 63.4  (56.8-69.5) | 88.7  (81.9-96.4) | 38.5  (32.4-44.8) | 97.5  (93.1-100.0) | 84.2  (79.3-93.5) | 93.8  (90.0-98.9) |

mNGS, metagenomic next-generation sequencing. CMT, conventional microbiological testing. PPV, positive predictive value. NPV, negative predictive value.
