# Supplementary material for: Diagnostic value of metagenomic next generation sequencing of bronchoalveolar lavage fluid in immunocompromised patients with pneumonia
Source: Front Cell Infect Microbiol. 2025 Sep 16;15:1602636. doi: 10.3389/fcimb.2025.1602636 (PMC12479419; doi:10.3389/fcimb.2025.1602636)
Supplement: Supplementary file 2 [file Table2.docx]

**Supplementary File2**

**Table 1. Specimen Collection**

| Specimen Types | Acquisition Methods | Types of Pathogens |
| --- | --- | --- |
| Lower respiratory secretions [1-3] | I. Expectoration Method  First rinse the mouth several times with cold boiled water. Then forcefully expel sputum from deep within the tracheobronchial tree. The specimen should be collected into a sterile container, with a minimum volume of 1 mL.  II. Bronchoscopy Aspiration Sampling  Under direct visualization with a fiberoptic bronchoscope, insert the suction catheter into the bronchus. Aspirate secretions from the pulmonary segment or subsegmental bronchus distal to the lesion site, and collect the secretions into a sterile container. | Bacteria, Fungus, Virus |
| Bronchoalveolar Lavage Fluid [3,4] | I. Preoperative Preparation  ①Assessment & Contraindications: Perform routine clinical evaluation to exclude contraindications, strictly adhering to bronchoscopy indications. ②Local Anesthesia: Use 2% lidocaine. Assess contraindications for intravenous anesthesia; use cautiously in elderly/debilitated patients or those with cardiac/pulmonary/hepatic/renal impairment. ③Monitoring: Continuous ECG and SpO₂ monitoring during procedure.  II. Lavage Procedure  ①Site Selection: Target affected segment for localized lesions; right middle lobe/left lingula for diffuse disease. ②Local Anesthesia: Instill 1-2 mL 2% lidocaine via biopsy port into target segment. ③Aspiration: After wedging bronchoscope tip at target subsegment, rapidly inject 60-120 mL sterile saline. Immediately aspirate with negative pressure <100 mmHg (adjust to prevent collapse). Minimum return rate: ≥30%.④BALF Collection: Collect in sterile containers. For large airway disease: process 1st aliquot separately. For non-large airway disease: pool aliquots. Submit 10–20 mL labeled specimens to microbiology lab within 2 hours. | Bacteria, Fungus, Virus |
| Sputum Culture [1,5,6] | I. Expectoration Method  Following oral rinsing with cold boiled water, collect more than 1 mL deep-cough sputum into a sterile container; qualify specimens exhibiting mucoid/purulent consistency.  II. Inoculation and Culture  Homogenize with sterile saline to distribute microorganisms uniformly. Within a biosafety cabinet, select appropriate culture media and inoculate using sterile tools under aseptic conditions. Incubate media at 35-37°C with humidity/CO₂ control: bacterial cultures for 18-24 hours, fungal for 48-72 hours, extending to more than 7 days for slow-growing pathogens. Regularly monitor colony morphology to preliminarily identify microbes. Conduct pathogen identification via Gram staining and biochemical reactions, performing antimicrobial susceptibility testing (AST) when clinically indicated. | Bacteria, Fungus, Virus |
| Blood Serum [7,8] | Under sterile conditions, a minimum of 3 mL venous blood is collected using pathogen-specific tubes: pyrogen-free serum separator tubes (gold-top) for G-test and GM-test, dedicated heparin sodium anticoagulant tubes (green-top) for tuberculosis T-cell detection, and EDTA anticoagulant tubes (purple-top) for viral testing; following collection, tubes are gently inverted 4–5 times to prevent clotting, with all whole blood samples transported to the laboratory within 8 hours. | Bacteria, Fungus, Virus |
| Oropharyngeal Swab [9] | Using a tongue depressor, the subject opens their mouth with soft palate elevation to expose the posterior pharyngeal wall. Insert a polyester swab, vigorously swab both tonsils (3 strokes each) and posterior pharynx in a Z-pattern (3 strokes) with ≥10s mucosal contact. Place in VTM, snap the shaft, vortex 10s. Seal labeled samples in biohazard bags; transport at 2-8°C if delayed >2h. | Virus |

**Table 2. CMT Methods**

| Detection Method | Procedure |
| --- | --- |
| G-Test [10,11] | Using sterile venipuncture, collect more than 3 mL serum. After 30-minute room-temperature clotting, centrifuge to obtain supernatant. Inactivate proteases by 37°C incubation for 10 minutes, then perform 1:10 dilutions with endotoxin-free water. Follow ELISA kit instructions for subsequent steps. Measure absorbance at 405 nm, calculate concentration against a standard curve, and interpret results more than 80 pg/mL as positive for fungal infection. |
| GM Test [10,12] | Aseptically collect 3 mL venous blood (draw before β-lactam antibiotic administration), allow 30-minute room-temperature clotting, and centrifuge to obtain serum. Perform ELISA according to kit instructions. Measure optical density at 450nm/620nm, calculate the index (sample OD / cutoff control OD), with a single serum index more than 0.5 interpreted as positive and BALF fluid index more than 1.0 considered positive. |
| Interferon gamma release assay [13] | Aseptically collect >4 mL venous blood into dedicated heparin tubes (green-top), immediately invert to mix, store at room temperature, and deliver to the lab within 8 hours; specimens undergo 16–24 hours of 37°C shaking incubation followed by centrifugation to harvest plasma; IFN-γ concentrations are measured via ELISA comparing antigen-stimulated wells with negative controls, where positive results indicate Mycobacterium tuberculosis infection though active TB and nontuberculous mycobacterial cross-reactivity require exclusion. |
| X-pert [14,15] | BALF samples were mixed with an equal volume of NALC-NaOH digestant, vortexed for 30 seconds, and incubated at room temperature for 15 minutes for liquefaction. A 1-mL aliquot of the processed sample was loaded into the X-pert cartridge. The system automatically performed ultrasonic lysis, solid-phase DNA extraction, and nested PCR amplification. Real-time fluorescence detection determined the presence of Mycobacterium tuberculosis complex DNA, bacterial load, and rifampicin resistance. |
| Viral Serological Testing [16-18] | Blood samples (≥3 mL) are collected aseptically. Herpes simplex virus (HSV) IgM/IgG antibodies are detected via immunity filtration assay for typing diagnosis. Epstein-Barr virus (EBV) testing employs chemiluminescence immunoassay (CLIA) to quantify VCA-IgM antibodies. Cytomegalovirus (CMV) screening utilizes ELISA for IgM/IgG antibody detection. All three assays require paired serum samples to enhance diagnostic accuracy. |
| Viral Throat Swab Testing [9] | The COVID-19 nucleic acid extraction via magnetic beads is performed in a biosafety cabinet. Real-time RT-PCR targets ORF1ab/N genes with RNase P internal control. Positivity requires: dual-target Ct≤37, S-shaped amplification curves, and validated negative controls.  Using Real-time RT-PCR detects Influenza M1 gene and Influenza B NS gene with human RNP internal control (IC). Positivity requires: target Ct≤35 (A) or ≤37 (B), S-shaped amplification curves, and validated negative controls. |

**Table 3. mNGS Methods**

| Detection Method | Procedure |
| --- | --- |
| Wet Lab [19,20] | Sample Collection: Bronchoalveolar lavage fluid (BALF) (≥5 mL) was collected into sterile DNase/RNase-free tubes pre-filled with nucleic acid preservation solution. Samples were immediately transported on ice and stored at 4°C for ≤72 hours.  DNA Extraction: Samples were lysed by adding protease K-containing lysis buffer (TIANamp Micro DNA Kit, Tiangen Biotech) and incubating at 56°C for 30 min. Subsequently, 0.1-mm zirconia beads were added, and mechanical disruption was performed at 30 Hz for 15 min using a tissue homogenizer. DNA was eluted with 50 μL of preheated (65°C) elution buffer TB and quantified using Qubit® 4.0 fluorometry.  Library Construction: Libraries were prepared using the Illumina® DNA Prep Kit. DNA was fragmented to achieve optimal insert sizes of 200–300 bp, followed by end repair, adenylation, adapter ligation, and PCR amplification.  High-Throughput Sequencing: Sequencing was performed on the MGISEQ-2000 platform, generating 20–30 million reads per sample to ensure sufficient depth for low-abundance pathogen detection. |
| Dry Lab [21,22] | Bioinformatic Analysis: Raw sequencing data underwent initial quality control (FastQC), followed by Trimmomatic to remove low-quality reads and adapter sequences. Host DNA was depleted by aligning reads to the human reference genome (hg19) using BWA [1]. High-quality microbial reads were taxonomically classified and quantified by parallel alignment to four curated microbial databases (viruses, bacteria, fungi, parasites) from NCBI (ftp://ftp.ncbi.nlm.nih.gov/genomes/). Low-complexity sequences and potential contaminants were filtered out.  Pathogens were reported only if meeting all four criteria:  (1) Minimum read thresholds (bacteria/fungi: ≥10; viruses: ≥5; parasites: ≥100)(2) Relative abundance >0.1% of total microbial reads;(3) Genome coverage ≥1% of reference length;(4) Consistency with clinical manifestations.  Borderline results (bacteria/fungi: 5–9; viruses: 2–4; parasites: 50–99) underwent triple verification: manual inspection of alignments, NT database BLASTn (e-value <1e−5), and comparison with same-batch negative controls. Pathogens with abundance ≥5-fold higher than controls were reported after screening against an internal contamination database. Antimicrobial resistance (AMR) genes were identified using the Comprehensive Antibiotic Resistance Database (CARD) (nucleotide identity ≥90%, coverage ≥80%), and virulence factors were analyzed via the Virulence Factor Database (VFDB) (≥5 supporting reads). |
| Quality Control, Ambiguous Result Handling [22] | Quality Control: Contaminant filtering employed a three-level control system (extraction/sequencing/reagent blanks) and a laboratory contamination database (pathogen reads ≥5× negative controls). Dynamic thresholds were applied: BALF samples required ≥5 reads (bacteria/fungi) or ≥3 reads (viruses), while non-sterile samples used threefold higher thresholds, with abundance thresholds adaptively adjusted by sequencing depth. Clinical relevance was quantified using a CRS scoring system (host immune status: 40%; inflammatory markers: 30%; imaging: 20%; treatment response: 10%). Special pathogens (e.g., Mycobacterium tuberculosis, Legionella, Nocardia, Aspergillus) underwent customized molecular marker validation with adjusted thresholds.  Ambiguous Result Handling: All borderline results required triple validation: Technical: raw data reanalysis and qPCR confirmation; Bioinformatic: genomic coverage profiling and marker gene localization; Clinical: consistency with antimicrobial susceptibility testing and treatment outcomes. Final reports were generated by multidisciplinary experts (including bioinformatics analysts, microbiologists, and clinical physicians) based on evidence levels (A–D) and confidence scores (0–100). |
| Clinical Report Generation | Report Generation: Positive results were processed through Beijing Genomics Institute (BGI)’s proprietary reporting system to generate standardized reports containing: (1) Patient demographics;(2) Pathogen list categorized as bacteria, fungi, DNA viruses, parasites, M. tuberculosis, and other microbes, annotated with read counts;(3) Metrics: total sequencing reads, human cell density (cells/mL), theoretical sensitivity, and detection sensitivity;(4) Annotations: assay limitations, potential colonizer alerts, and retest recommendations. Turnaround time from sample receipt to report delivery was 24–48 hours. |

**References：**

1. Joyce SM. Sputum analysis and culture. Ann Emerg Med. 1986 Mar;15(3):325-8.

2. Lode H, Schaberg T, Raffenberg M, Mauch H. Diagnostic problems in lower respiratory tract infections. J Antimicrob Chemother. 1993 Jul;32 Suppl A:29-37.

3. Meyer KC, Raghu G, Baughman RP, Brown KK, Costabel U, et al; American Thoracic Society Committee on BAL in Interstitial Lung Disease. An official American Thoracic Society clinical practice guideline: the clinical utility of bronchoalveolar lavage cellular analysis in interstitial lung disease. Am J Respir Crit Care Med. 2012 May 1;185(9):1004-14.

4. Sun W, Zheng L, Kang L, Chen C, Wang L, Lu L, Wang F. Comparative analysis of metagenomic and targeted next-generation sequencing for pathogens diagnosis in bronchoalveolar lavage fluid specimens. Front Cell Infect Microbiol. 2024 Aug 27; 14:1451440.

5. Losier A, Dela Cruz CS. New testing guidelines for community-acquired pneumonia. Curr Opin Infect Dis. 2022 Apr 1;35(2):128-132.

6. File TM Jr. Case studies of lower respiratory tract infections: community-acquired pneumonia. Am J Med. 2010 Apr;123(4 Suppl): S4-15.

7. Mikulska M, Furfaro E, Viscoli C. Non-cultural methods for the diagnosis of invasive fungal disease. Expert Rev Anti Infect Ther. 2015 Jan;13(1):103-17.

8. Suárez I, Fünger SM, Kröger S, Rademacher J, Fätkenheuer G, Rybniker J. The Diagnosis and Treatment of Tuberculosis. Dtsch Arztebl Int. 2019 Oct 25;116(43):729-735.

9. Di Maio P, Iocca O, Cavallero A, Giudice M. Performing the nasopharyngeal and oropharyngeal swab for 2019-novel coronavirus (SARS-CoV-2) safely: How to dress, undress, and technical notes. Head Neck. 2020 Jul;42(7):1548-1551.

10. Hage CA, Carmona EM, Epelbaum O, Evans SE, Gabe LM, Haydour Q, et al. Microbiological Laboratory Testing in the Diagnosis of Fungal Infections in Pulmonary and Critical Care Practice. An Official American Thoracic Society Clinical Practice Guideline. Am J Respir Crit Care Med. 2019 Sep 1;200(5):535-550.

11. Lamoth F, Alexander BD. Nonmolecular methods for the diagnosis of respiratory fungal infections. Clin Lab Med. 2014 Jun;34(2):315-36.

12. Shin B, Koh WJ, Jeong BH, Yoo H, Park HY, Suh GY, et al. Serum galactomannan antigen test for the diagnosis of chronic pulmonary aspergillosis. J Infect. 2014 May; 68(5):494-9.

13. Ontario Health. Interferon-Gamma Release Assay Testing for Latent Tuberculosis Infection: A Health Technology Assessment. Ont Health Technol Assess Ser. 2024 Dec 12;24(11):1-183.

14. Bodmer T, Ströhle A. Diagnosing pulmonary tuberculosis with the Xpert MTB/RIF test. J Vis Exp. 2012 Apr 9;(62): e3547.

15. Horne DJ, Kohli M, Zifodya JS, Schiller I, Dendukuri N, Tollefson D, Schumacher SG, Ochodo EA, Pai M, Steingart KR. Xpert MTB/RIF and Xpert MTB/RIF Ultra for pulmonary tuberculosis and rifampicin resistance in adults. Cochrane Database Syst Rev. 2019 Jun 7;6(6):CD009593.

16. Wald A, Ashley-Morrow R. Serological testing for herpes simplex virus (HSV)-1 and HSV-2 infection. Clin Infect Dis. 2002 Oct 15;35(Suppl 2): S173-82.

17. Niller HH, Bauer G. Epstein-Barr Virus: Clinical Diagnostics. Methods Mol Biol. 2017; 1532:33-55.

18. Lazzarotto T, Guerra B, Lanari M, Gabrielli L, Landini MP. New advances in the diagnosis of congenital cytomegalovirus infection. J Clin Virol. 2008 Mar;41(3):192-7.

19. Lopez-Labrador FX, Huber M, Sidorov IA, Brown JR, Cuypers L, Laenen L, et al; European Society of Clinical Virology (ESCV) Network on Next-Generation Sequencing (ENNGS). Multicenter benchmarking of short and long read wet lab protocols for clinical viral metagenomics. J Clin Virol. 2024 Aug; 173:105695.

20. Gu W, Miller S, Chiu CY. Clinical Metagenomic Next-Generation Sequencing for Pathogen Detection. Annu Rev Pathol. 2019 Jan 24; 14:319-338.

21. Roy S, Coldren C, Karunamurthy A, Kip NS, Klee EW, Lincoln SE, et al. Standards and Guidelines for Validating Next-Generation Sequencing Bioinformatics Pipelines: A Joint Recommendation of the Association for Molecular Pathology and the College of American Pathologists. J Mol Diagn. 2018 Jan;20(1):4-27.

22. Gargis AS, Kalman L, Lubin IM. Assuring the Quality of Next-Generation Sequencing in Clinical Microbiology and Public Health Laboratories. J Clin Microbiol. 2016 Dec;54(12):2857-2865.
